# Radiation cancer risk at different dose rates: new dose-rate effectiveness factors derived from revised A-bomb radiation dosimetry data and non-tumor doses

**DOI:** 10.1093/jrr/rrab109

**Published:** 2021-12-20

**Authors:** Hiroshi Tanooka

**Keywords:** radiation, cancer, dose rate, non-tumor dose, dose-rate effectiveness factor (DREF)

## Abstract

The dose rate of atomic bomb (A-bomb) radiation to the survivors has still remained unclear, although the dose–response data of A-bomb cancers has been taken as a standard in estimating the cancer risk of radiation and the dose and dose-rate effectiveness factor (DDREF). Since the applicability of the currently used DDREF of 2 derived from A-bomb data is limited in a narrow dose-rate range, 0.25-75 Gy/min as estimated from analysis of DS86 dosimetry data in the present study, a non-tumor dose (D_nt_) was applied in an attempt to gain a more universal dose-rate effectiveness factor (DREF), where D_nt_ is an empirical parameter defined as the highest dose at which no statistically significant tumor increase is observed above the control level and its magnitude depends on the dose rate. The new DREF values were expressed as a function of the dose rate at four exposure categories, i.e. partial body low LET, whole body low linear energy transfer (LET), partial body high LET and whole body high LET and provided a value of 14 for environmental level radiation at a dose rate of 10^−9^ Gy/min for whole body low LET.

## INTRODUCTION

The dose–response relation is essential for the quantitative expression of radiation- induced cancer risk. However, there are two different expressions in the dose–response curve. One is a plot of the cancer incidence rate as a function of radiation dose given at a fixed dose rate with varying exposure times. Experimental data are often expressed in this way. The other expression is a plot of the cancer incidence-rate against the dose given with varying dose rates at a fixed exposure time. The A-bomb cancer data belong to this type.

The A-bomb data from the life span study (LSS) have been widely applied as a standard for evaluation of the cancer risk of radiation and the dose-rate effects. This raises the question of what the dose rate of A-bomb radiation is. The question is simple; the answer is not easy. The extensive studies on dosimetry of A-bomb radiation were conducted by elaborative measurements and calculations [1, 2]. The dose rate was analyzed for each component of the A-bomb radiation in detail [3]; however, the dose rates to the survivors on the ground at different distances from the hypocenter still remain undetermined. The time duration for the nuclear fission is 1 μsec. When this value is applied to the exposure time of the A-bomb radiation to the survivors, it becomes an overestimation of the dose rate such as 1 Gy/μsec or 6 x 10^7^ Gy/min for a median dose of 1 Gy. This point is vital in applying the A-bomb data as a standard to evaluate the radiation cancer risk. Hence, a more realistic estimation of the dose-rate to the survivors is needed.

On the other hand, epidemiological studies have been extensively conducted at the Radiation Effects Research Foundation (RERF), as summarized by Ozasa *et al.* [4]. The LSS study at RERF included cancer mortality data on leukemia [5–7] and solid cancers [8–10]. To analyze the dose-rate effect, the dose–response curves for these cancers were fitted to a linear-quadratic model [11–13]. The early history of determination of the dose-rate effectiveness factor (DREF) up to 1990 was summarized by Fabricant [14]. The analysis of the dose-rate effects on A-bomb data together with experimental animal data was conducted by ICRP, NCRP, NRPB and UNSCEAR committees [15–20], giving the dose and dose-rate effectiveness factor (DDREF) in the range of 1.4–10. Currently, a DDREF of 2 is applied in practice for the radiation protection purpose as recommended by ICRP and BEIR [15, 16]. However, an increasing number of evidences exist for a higher DDREF value from human and experimental animal data. For example, a high value of the DREF, as high as 35, was presented from lung cancer data with internal low LET radiation emitters inhaled to dogs [21]. Such a discrepancy is yet to be explained. Here in this review, the term DREF is adopted for an expression of the degree of the dose-rate effects, since its definition and the method of its derivation are distinguished from DDREF [21].

Since the shape of the dose–response curve for radiation-induced cancers varies depending upon the dose-rate of radiation, the radiation risk cannot be evaluated from the slope of the dose–response curve in many cases. For universal expression of the radiation cancer risk, the idea of a non-tumor dose (D_nt_) was introduced in previous studies [22–25], where D_nt_ is an empirical factor defined as the highest dose at which no statistically significant tumor increase is observed above the control level and surveyed in the literature. The collected D_nt_ values were expressed as a function of the dose rate of radiation in the four exposure categories. The D_nt_ can be applied to any exposure conditions as a dose-rate dependent indicator of radiation cancer risk. In this study, the DREF values were obtained from the ratio of D_nt_ at respectively different exposure conditions to the D_nt_ of A-bomb cancers.

This study professes no extensive review of the literature; what it hopes to do is to emphasize the problems involved in the assessment of the dose-rate effect on the radiation cancer risk and attempts to provide a more widely applicable indicator of the dose-rate effect.

## DOSE RATE AND DDREF OF A-BOMB RADIATION

The A-bomb data has been used as a standard to estimate the dose-rate effect on the radiation cancer risk. Therefore, it is essentially important to determine the dose rate of A-bomb radiation to the survivors who received different doses at different distances from the hypocenter. As the first step, the average exposure time was estimated from the DS86 dosimetry data. Table 1 shows various components of A-bomb radiations, i.e. γ rays and neutrons [26]. Table 2 shows that γ rays were the major component to contribute to the total absorbed dose [27], which is in agreement with analysis of Rühm *et al.* [3]. Nuclear fission occurs within 1 μsec after detonation of the A-bomb. However, application of 1 μsec for the exposure time results in an overestimation of the dose rate to the survivors [3, 22, 23].

**Table 1:** Source of ionizing radiation from a nuclear weapon (DS86, Ref. 26). Quoted with permission of the publisher

Source	Time emitted after detonation
Prompt neutrons from fission	< 1 μsec
Delayed neutrons from fission products	< 1 min
Prompt γ rays from fission process	< 1 μsec
γ rays from inelastic scattering	
From weapon	< 1 μsec
From air	< 10 μsec
From ground	< 10 μsec
γ rays from charged particle reactions	
From weapon	< 1 μsec
From air	< 10 μsec
From ground	< 10 μsec
Capture γ rays	
From weapon	< 1 μsec
From air	Few msec to 0.2 sec
From ground	Few msec to 0.2 sec
Activation γ rays	
Early time	0.2 sec to 1 min
Residual	1 min to years
Delayed γ rays from fission products	
Early time	0.2 sec to 1 min
Residual	1 min to years

**Fig. 1:**
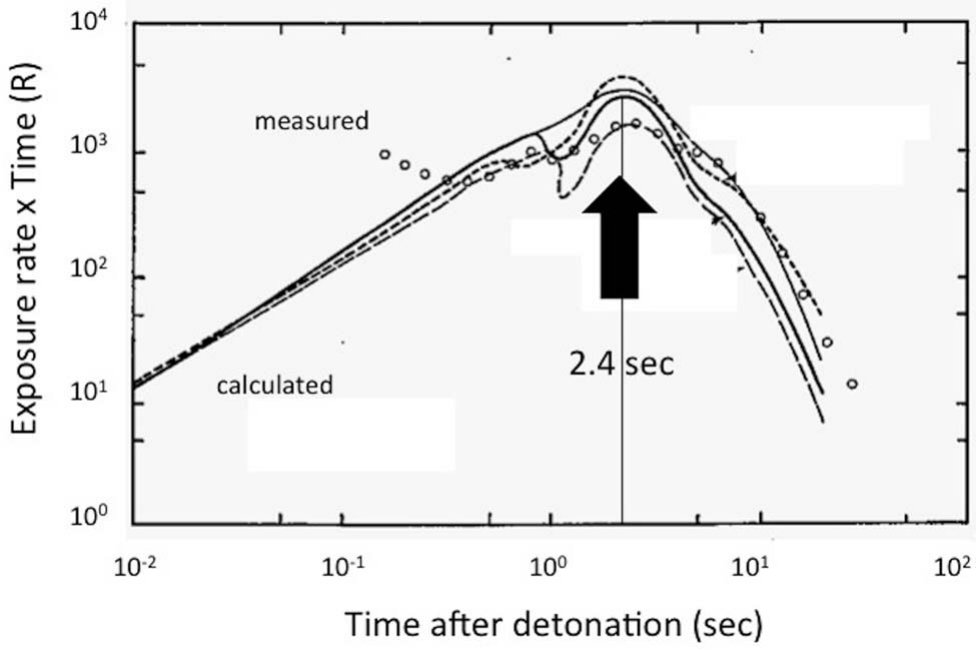
Variation of the dose rate of A-bomb radiation at a distance of 914 m from the hypocenter on the ground after detonation of the bomb, as expressed by the exposure rate multiplied by time (in R unit) in the DS86 data (Fig. 16 in Ref. 26). The time at a peak dose-rate, 2.4 sec, was used as the average exposure time of A-bomb radiation for estimation of the dose rate. Quoted with permission of the publisher.

**Fig. 2:**
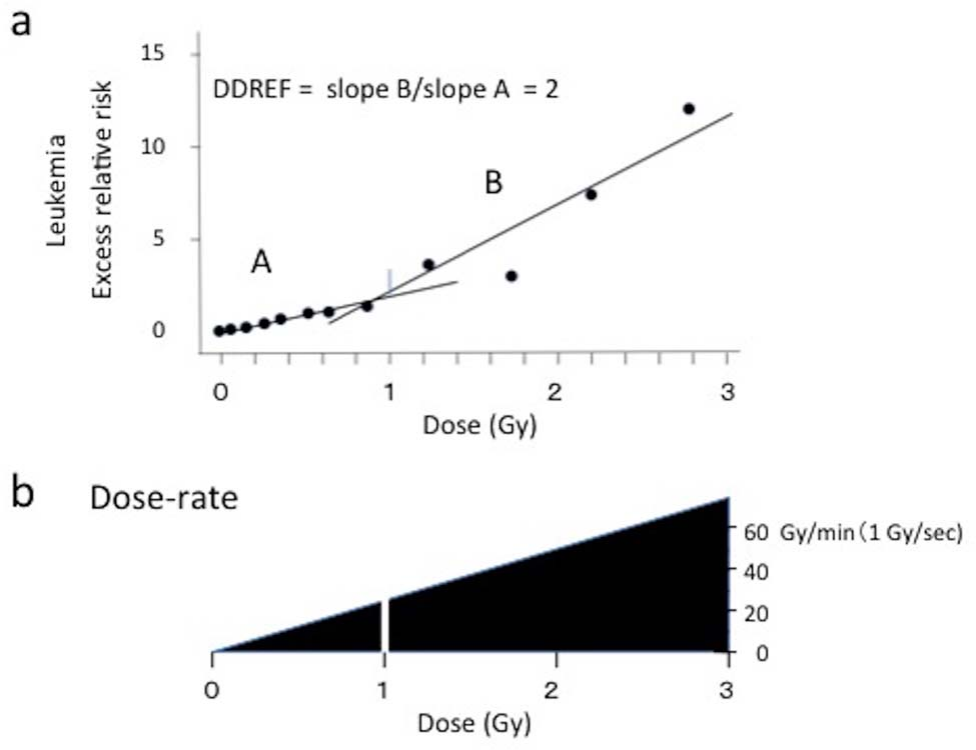
(a) Schematic expression of the dose–response relation of A-bomb leukemia data [7] and determination of DDREF from a single dose–response relation. Cancer data were divided into low and high dose regions and two straight lines were applied to estimate the cancer incidence rate by comparison of the slope of the line. The ratio of the two slopes yielded DDREF = 2. (b) The varying dose rate of A-bomb radiation corresponding to respective doses to the survivors, calculated by applying the average exposure time, 2.4 sec.

To gain a more realistic dose rate, the exposure time of A-bomb radiation to the survivors was estimated from the results of the Nevada experiments and calculations [26]. Fig. 1 shows a time course of the delivery of the radiation dose at a distance of 914 m from the hypocenter after detonation of the bomb in terms of the exposure rate (in Ref. 26). From this figure, it became clear that the radiation dose was delivered within 20 sec, with a peak at 2.4 sec after detonation of the bomb. This peak appeared at 3 sec at a distance of 2779 m (in Ref. 26). In the present analysis, the peak exposure time of 2.4 sec was taken as an average for the whole A-bomb radiation to the survivors. Accordingly, the dose rate to the survivors was calculated by dividing the absorbed dose, shown in the axis of the dose–response curves for A-bomb cancers, by the average exposure time 2.4 sec. Fig. 2a shows the dose–response data for A-bomb leukemia [7]. It should be noted that the doses shown in the axis of the dose–response figure represent the dose given at different dose-rates. This variation is explicitly shown in Fig. 2b. At a dose of 1 Gy for example, the dose rate was 1 Gy/2.4 sec = 0.42 Gy/sec or 25 Gy/min, apart from the dose-rate estimated from the assumption of 1 μsec for the exposure time, 10^6^ Gy/sec or 6 x 10^7^ Gy/min. It should be noted that the dose rate of low LET radiation at the environmental level is as low as 10^−9^-10^−8^ Gy/min.

**Table 2:** Contribution of each component of A-bomb radiation to the total absorbed dose at the ground level analyzed by Imanaka (Ref. 27). Quoted with permissions of the author and publisher

Radiation	Effective exposure time	Contribution
Prompt radiation		
Primary γ rays	< 1 μsec	●●●
Neutrons	< 1 msec	●●
Secondary γ rays	< 0.1 sec	●●●●
Delayed radiation		
γ rays	< 30 sec	●●●●
Neutrons	< 10 sec	●
Secondary γ rays	< 10 sec	●
Residual radiation	months	●●

One may wonder how the dose-rate effect can be determined from a single dose–response curve. This question can be answered from the fixed exposure time of radiation that delivered different dose rates at different distances, so that the low dose region represents low dose-rates and the high dose region represents high dose-rates.

An early estimation of DDREF of 2 was obtained by comparing the slopes of the two lines applied to measured points at the low dose region with the slope at the high dose region (Fig. 2a) [15]. Further, the linear-quadratic or the combination of linear and quadratic model was applied to estimate DDREF. The present study showed that the DDREF value of 2 currently applied to radiation protection covers a relatively narrow dose-rate range, 0.25-75 Gy/min for the dose range of the A-bomb cohort study, 0.01-3 Gy.

## **NON-TUMOR DOSE** (${\bf D}_{\bf NN} $) AS A MEASURE OF RADIATION CANCER RISK

Since the shape of the dose–response curve for radiation cancer incidence varies with varying dose rates, a universal indicator of the radiation cancer risk is needed. The slope of the dose–response curve can be the indicator when the shape of the curve is linear. UNSCEAR surveyed the lowest doses that yield a significant increase in the cancer incidence [20]. This indicator is useful for estimating the cancer risk for various types of the dose–response curve, although statistical consideration is needed. In the present study, the idea of a non-tumor dose, D_nt_, defined as the highest dose of radiation that produces no tumor above the control level, was applied as another indicator. The idea of D_nt_ was introduced in the French textbook *Radiobiologie* by Tubiana [28]. Although numerous reports showed the dose–response relation for radiation induced cancers, not many reports showed the non-tumor dose region with a known dose rate. Selected data [22, 23] consisted of external and internal exposure cases. Internal exposure cases were particularly useful for providing D_nt_ values at a low dose-rate range. The analysis combined 56 human and animal data and all types of tumors together.

For human cancers, the D_nt_ value was 0.1 Gy for A-bomb solid cancers (whole body, low LET) at a dose rate of 2.5 Gy/min estimated from the published data [8–10] according to the definition of D_nt_. Other D_nt_ values for human cancers were 10 Gy for bone tumors in radium painters (partial body high LET) [29], 2 Gy for liver tumor in thorotrast-injected patients (partial body high LET) [30] and 1 Gy for soft tissue tumors after radiation therapy (partial body low LET) [31]. The absence of a cancer increase in residents in the high natural radiation background area (whole body low LET) in India [32] and in China [33] indicates a high D_nt_ value at a low-dose rate.

For experimental animal cancers, a threshold-like dose response is found in many cases. Acute whole body γ-irradiation of mice with a total dose of 7.2 Gy in four repeats produced thymic lymphoma with a frequency of 90%, while no tumor was produced with the same total dose irradiated at a low-dose rate, 2 x 10^−5^ Gy/min (D_nt_ > 7.2 Gy) [34]. Such a threshold-like dose–response is found in the tumor incidence in mouse skin irradiated repeatedly with β-rays (partial body low LET) [35, 36]. Internal emitter experiment data are particularly useful to study the low dose-rate effects, as seen in earlier studies [37]. Oral administration of tritiated water into mice (whole body low LET) produced no lymphoma with a threshold dose-rate of 6.4 x 10^−7^ Gy/min (D_nt_ = 0.71 Gy) [38]. A high LET radiation has been considered to have no dose-rate effect in cancer incidence. However, at a very low dose rate, α-particles of ^222^Rn inhaled into the rat lung (partial body high LET) gave a much lower tumor incidence than in rats given the same total dose at a high-dose rate [39].

**Fig. 3:**
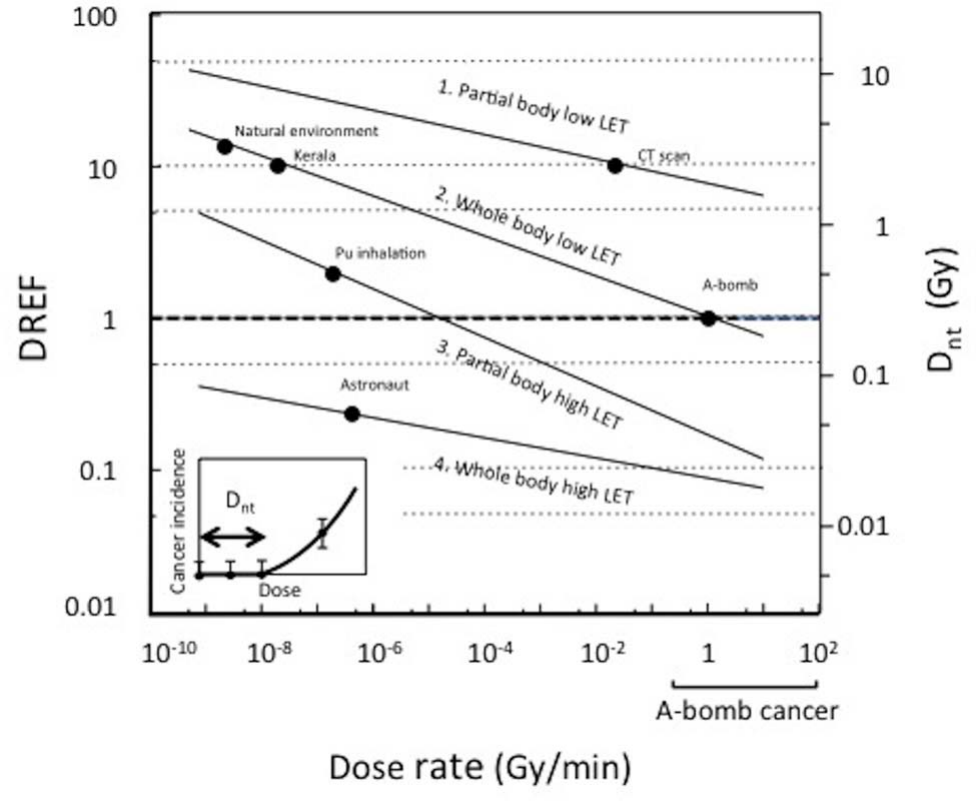
DREF expressed as a function of the dose rate of radiation in the four exposure categories corresponding to equations 2-1~2-4 (scale on the left ordinate) by normalizing the regression lines for D_nt_ (scale on the right ordinate). Estimation of D_nt_ is shown at the lower left corner. The range of the dose rate of A-bomb radiation covered in the cancer cohort study is shown at the lower right corner. ●: human exposure cases.

Altogether, these D_nt_ values were divided into four categories and plotted as a function of the dose rate with regression lines applied to data points [23], i.e.

(1–1) \begin{equation*} {\mathrm{D}}_{\mathrm{nt}}=2.69{\mathrm{X}}^{-0.0857},\kern1.33em {\mathrm{R}}^2=0.147,\kern0.33em \mathrm{for}\ \mathrm{partial}\ \mathrm{body}\ \mathrm{low}\ \mathrm{LET} \end{equation*}

(1–2) \begin{equation*}\! \! \! {\mathrm{D}}_{\mathrm{nt}}=0.25{8\mathrm{X}}^{-0.141},\kern1em {\mathrm{R}}^2=0.320,\kern0.33em \mathrm{for}\ \mathrm{whole}\ \mathrm{body}\ \mathrm{low}\ \mathrm{LET} \ \ \end{equation*}

(1–3) \begin{equation*} {\mathrm{D}}_{\mathrm{nt}}=0.0439{\mathrm{X}}^{-0.167},\kern.7em {\mathrm{R}}^2=0.303,\kern0.33em \mathrm{for}\ \mathrm{partial}\ \mathrm{body}\ \mathrm{high}\ \mathrm{LET}\quad\quad \qquad\end{equation*}

(1–4) \begin{equation*} \ {\mathrm{D}}_{\mathrm{nt}}=0.0207{\mathrm{X}}^{-0,0733},\kern.5em {\mathrm{R}}^2=0.781,\mathrm{for}\ \mathrm{whole}\ \mathrm{body}\ \mathrm{high}\ \mathrm{LET},\qquad \quad\end{equation*}

where X is dose rate in Gy/min. Using the above equations, one can find D_nt_ values corresponding to any possible radiation exposure condition. The regression lines are shown in Fig. 3 for D_nt_ values on the right ordinate.

## NEW DREF VALUES DERIVED FROM NON-TUMOR DOSES

The non-tumor dose D_nt_ is a dose-rate dependent indicator for radiation cancer risk.

Accordingly, new DREF values were estimated from the ratio of the D_nt_ values for respective exposure conditions to the D_nt_ for the A-bomb radiation as a standard. The derivation of DREF was obtained by normalizing equations for Dnt 1-1 ~ 1-4 so that the DREF for the A-bomb solid cancer becomes 1. Dnt for A-bomb solid cancers is 0.1 Gy at the dose rate of 2.5 Gy/min read from the original data or 0.26 Gy at the same dose rate estimated from equation 2-2 or the regression line for whole body low LET. The latter value was taken for normalization, by multiplying a factor of 1.3776 to equation 2-1~2-4, so that DREF is 1 at the dose rate of 2.5 Gy/min for whole body low LET. The shape of the regression lines was not changed by this conversion. New DREF values were expressed as a function of the dose-rate as follows:

(2–1) \begin{equation*} {\mathrm{DREF}}_{\mathrm{PL}}=11.85{\mathrm{X}}^{-0.0857},\kern2em \mathrm{for}\ \mathrm{partial}\ \mathrm{body}\ \mathrm{low}\ \mathrm{LET} \ \ \ \ \quad \end{equation*}

(2–2) \begin{equation*} {\mathrm{DREF}}_{\mathrm{WL}}=1.13{8\mathrm{X}}^{-0.141},\kern2em \mathrm{for}\ \mathrm{whole}\ \mathrm{body}\ \mathrm{low}\ \mathrm{LET}\quad \ \ \ \ \end{equation*}

(2–3) \begin{equation*} {\mathrm{DREF}}_{\mathrm{PH}}=0.15{9\mathrm{X}}^{-0.167},\kern2em \mathrm{for}\ \mathrm{partial}\ \mathrm{body}\ \mathrm{high}\ \mathrm{LET}\quad \ \ \end{equation*}

(2–4) \begin{equation*} {\mathrm{DREF}}_{\mathrm{WH}}=0.0909{\mathrm{X}}^{-0,0733},\kern2em \mathrm{for}\ \mathrm{whole}\ \mathrm{body}\ \mathrm{high}\ \mathrm{LET}, \ \ \end{equation*}

where X is dose rate in Gy/min. Fig. 3 shows four lines corresponding to the above equations in the four exposure categories. All possible radiation exposure conditions are included in this figure. Examples of DREF values corresponding to various human exposure conditions are shown on the four lines. The new DREF values ranged from 0.1 to 100, depending on the dose rate from 10^−9^ to 10^2^ Gy/min.

The absence of a cancer increase in residents in the high natural radiation background area in India and in China indicates a high DREF value at a low-dose rate. A DREF of 10-14 would be an appropriate estimate for an elevated low LET radiation background level. Application of a DDREF of 2 to the diagnostic X-rays [40] overestimates the cancer risk as high as a 4.4 attributable cancer risk in the Japanese population, where a DREF of 10 should be applied for partial body low LET, assuming the linear dose response. In contrast, for astronauts in space flight (whole body high LET), a low DREF value is suitable.

## DISCUSSION

The two important findings in early studies on experimental radiation carcinogenesis with mice are the dose-rate effect on leukemia [41] and the whole body and partial body difference in the thymic lymphoma incidence [42]. These effects are expected to be present in humans, too. The two findings are recognized as the basis of the present study.

The present study showed an estimation of the radiation risk from new DREF values derived from the non-tumor dose, D_nt_, as an extension of the previous analysis [22, 23], where D_nt_ values were expressed as a function of the dose rate of radiation. The original analysis included the dose–response cancer data on humans and experimental animals, i.e. mice, rats and dogs and various organs and types of tumors together. Further, external and internal exposure cases were mixed on the basis of the absorbed dose. For the partial body exposure, calibration of the mass of the target organ or tissue may have to be considered [30]. From these points, the present estimating method is considered to be still incomplete. However, D_nt_ values can be estimated without considering the shape of the dose–response curve even for the linear-type dose response according to its definition.

DREF is an indicator of cancer risk differences and does not directly include the effect of the dose. To obtain the magnitude of the cancer risk at a given dose by extrapolating the A-bomb data to a different dose-rate, DREF can be used only when the dose response is linear at the corresponding dose rate. This quantitative estimation will be possible for a high-dose rate for whole body low LET and high LET radiations. However, for low-dose rate of whole body LET and partial body low LET the shape of the dose–response curve is not known exactly, although the threshold type is expected. Ideally, dose–response relations for each individual dose rate are desirable. Furthermore, a recently developing field of new radiation therapy, flash radiation [43], will be included in the high dose-rate problem.

Brooks *et al.* presented a very high value of DREF, 35, from experiments with dogs inhaled with β and γ emitters (partial body low LET) [21]. This high DREF value can be explained by the partial body exposure at the low-dose rate. As an exception, the Techa River data on human leukemia [44], where the main source of radiation is external γ-rays from Sr^89^ in soil sediments, showed a dose–response similar to the A-bomb data, although the dose rate was much lower.

As an example of the application of the D_nt_ method to quickly estimate the cancer risk of radiation, a ^239^PuO_2_ inhalation accident that occurred at the Japan Atomic Energy Agency in June, 2017 (partial body high LET) was analyzed. The exposure condition is classified under the category of partial body high LET, since α-emitters were deposited into the lung. The amount of deposited radioactivity was 22 000 Bq and the estimated absorbed dose was 1.2 Sv/year assuming RBE of 20 (0.06 Gy/year or 1.14 x 10^−7^ Gy/min). If the calculation followed the ICRP method to estimate the cancer risk from this accident, i.e. 0.5% cancer increase per 100 mSv with DDREF of 2, the expected excess cancer risk is 3% for a yearly dose, or 30% for 10-year dose without consideration of decay. A DDREF of 2 is plausible because this is the case in partial body high LET exposure (Fig. 3). On the other hand, the D_nt_ value was estimated as 0.63 Gy from the equation 1-3. This estimation indicates that the yearly exposed dose is 10 fold lower than the corresponding D_nt_, or reaches the D_nt_ after 10 years without consideration of a half-life.

Finally, the present method is still crude and further to be improved in order to obtain a more clear correlation between the dose rate and DREF by adding new data. However, this method covers a possible wide dose-rate region and possible exposure conditions and will be useful for a rough estimation of the radiation cancer risk in the human environment.

## SUMMARY

The dose rate of A-bomb radiation to the survivors was estimated from the average exposure time, 2.4 sec and absorbed doses, as being 25 Gy/min for a dose of 1 Gy at the ground level. The dose-rate covered by the A-bomb cohort study appeared to be limited inside a relatively narrow range, 0.25-75 Gy/min, for the doses of 0.01-3 Gy. Non-tumor dose, D_nt_, was applied to obtain DREF. New DREF values ranged from 0.1 to 100, depending on the dose rate from 10^−9^ to 10^2^ Gy/min in the four exposure categories. An apparently large discrepancy in the radiation cancer risk can be explained by the difference in the dose rate and the exposure condition, i.e. whole body versus partial body. The radiation cancer risk at low-dose rates was estimated to be much lower than the risk estimated by a currently adopted DDREF value of 2.
